# Dosimetry of Submandibular Glands on Xerostomia for Nasopharyngeal Carcinoma

**DOI:** 10.3389/fonc.2020.601403

**Published:** 2021-01-11

**Authors:** Xin-Bin Pan, Yang Liu, Shi-Ting Huang, Su Pei, Kai-Hua Chen, Song Qu, Ling Li, Xiao-Dong Zhu

**Affiliations:** Department of Radiation Oncology, Guangxi Medical University Cancer Hospital, Nanning, China

**Keywords:** nasopharyngeal carcinoma, xerostomia, submandibular glands, NPC, intensity-modulated radiotherapy

## Abstract

**Purpose:**

To investigate dosimetry of submandibular glands on xerostomia after intensity-modulated radiotherapy for nasopharyngeal carcinoma (NPC).

**Methods:**

From September 2015 to March 2016, 195 NPC patients were investigated. Xerostomia was evaluated at 12 months after treatment *via* the RTOG/EORTC system. The least absolute shrinkage and selection operator regression model was used to optimize feature selection for grades 2–3 xerostomia. Multivariable logistic regression analysis was applied to build a predicting model incorporating the feature selected in the least absolute shrinkage and selection operator regression model. Discrimination, calibration, and clinical usefulness of the predicting model were assessed using the C-index, calibration plot, and decision curve analysis.

**Results:**

The V30 of the parotid glands was selected based on the least absolute shrinkage and selection operator regression. The nomogram displayed good discrimination with a C-index of 0.698 (95% confidence interval [CI]: 0.626–0.771) and good calibration (model 1). Addition of the dosimetric parameters including the mean dose to the submandibular glands, V50 of the submandibular glands, and volume of the submandibular glands to the model 1 failed to show incremental prognostic value (model 2). The model 2 showed a C-index of 0.704 (95% CI: 0.632–0.776). Decision curve analysis demonstrated that the model 1 was clinically useful when intervention was decided at the possibility threshold of > 20%. Within this range, net benefit was comparable between the model 1 and model 2.

**Conclusion:**

PGv30 was a major predictive factor of grades 2–3 xerostomia for NPC. In contrast, the mean dose to the submandibular glands, V50 of the submandibular glands, and volume of the submandibular glands were not independent predictive factors.

## Introduction

Nasopharyngeal carcinoma (NPC) is a radiosensitive cancer, which is high incidence in Southern China (1, 2). Radiation-induced xerostomia is a common complication after intensity-modulated radiotherapy (IMRT) (3). Up to 30% patients suffer from clinically significant xerostomia, which degrades patients’ quality of life (4, 5). Parotid glands produce 60%–65% of salivary output, while submandibular glands contribute 20%–30% of the salivary output (6, 7). Previous studies reported that mean dose to the parotid glands was a major predictor of xerostomia (8–13). However, dosimetry of submandibular glands on xerostomia for NPC was not well investigated. This study was conducted to identify dosimetric parameters of submandibular glands on xerostomia in NPC patients receiving IMRT.

## Materials and Methods

### Patients

This longitudinal study included newly pathologic confirmed NPC treated at Guangxi Medical University Cancer Hospital from September 2015 to March 2016. The inclusion criteria included the following: 1) World Health Organization type II or III; 2) stage I-IVb according to the 7^th^ edition of the American Joint Committee on Cancer; and 3) patients received IMRT. Exclusion criteria were as follows: 1) patients with heart failure, uncontrolled diabetes, severe hepatitis, or renal dysfunction; 2) patients did not complete radiotherapy; 3) patients with a follow-up time < 1-year; 4) patients with diseases that affected the secretion of salivary glands. This study was approved by Guangxi Medical University Cancer Hospital Ethics Committee.

### Radiotherapy

All patients received radical IMRT. Patients in the supine position were fixed with the head-neck-shoulder thermoplastic mask. The computed tomography simulation (CT-sim) scanned from the skull base to the sternal angle with a thickness of 2.5 mm. The gross tumour volume of the nasopharynx (GTVnx) and gross tumour volume of the cervical lymph nodes (GTVnd) were quantified by using computed tomography and magnetic resonance imaging scans. The high-risk clinical target volume (CTV1) included the GTVnx plus a 5–10 mm margin. The low-risk clinical target volume (CTV2) included the GTVnd, the lymphatic regions, and the CTV1 with 5–10 mm margins. The planning target volume (PTV) was defined by adding a 3 mm margin to the GTV or CTV.

The radiotherapy prescription dose was PGTVnx 70.06–72.32 Gy/31~32 f, PGTVnd 66.00–72.32 Gy/30~32 f, PCTV1 60.00–62.00 Gy/30~31 f, and PCTV2 54.00–55.80 Gy/30~31 f, respectively. The maximum dose of the brain stem, optic nerves, and chiasma were 54 Gy. The maximum dose of spinal and lens were 45 Gy and 7 Gy, respectively. V30 of the parotid glands was constrained to less than 50%. No dose constraint was given for the submandibular glands during optimization of all IMRT plans. All plans were step-and-shoot IMRT of nine fields.

### Chemotherapy

Cisplatin (100 mg/m^2^) every 3 weeks was used for concurrent chemotherapy during radiotherapy. Induction chemotherapy included three cycles of docetaxel (60 mg/m²) on day 1, cisplatin (60 mg/m^2^) on day 1 and 5-fluorouracil (600 mg/m^2^) daily for 5 consecutive days every 3 weeks. Of the 195 patients, 20 patients received radiotherapy alone, 175 patients received concurrent chemoradiotherapy with or without induction chemotherapy.

### Dosimetric Parameters

All the parotid glands and submandibular glands were contoured based on the CT-Sim. No margin was added during treatment planning for the parotid glands and submandibular glands. The dosimetric parameters were calculated from the dose-volume histograms in the radiotherapy planning system of Pinnacle³ 9.8 (Philips Co., Eindhoven, Netherlands). The dosimetric parameters included the mean dose to the submandibular glands (SMGmean), V50 of the submandibular glands (SMGv50), volume of the submandibular glands (SMGvolume), mean dose to the parotid glands (PGmean), V30 of the parotid glands (PGv30), V50 of the parotid glands (PGv50), and volume of the parotid glands (PGvolume).

### Xerostomia Evaluation

Xerostomia were assessed at 12 months after radiotherapy. Xerostomia was evaluated according to the Radiation Therapy Oncology Group/European Organization for Research and Treatment of Cancer (RTOG/EORTC) system (14).

### Statistical Analysis

SMGmean, SMGv50, SMGvolume, PGmean, PGv30, PGv50, PGvolume, and weight loss rate were expressed as the mean ± standard deviation. Differences of SMGmean, SMGv50, SMGvolume, PGmean, PGv30, PGv50, PGvolume, and weight loss rate between grades 0–1 and grades 2–3 xerostomia were compared using Student’s t-test or Mann-Whitney U test.

The least absolute shrinkage and selection operator (LASSO) method was used to select the optimal predictive factors predicting grade 2-3 xerostomia (15). The variables including SMGmean, SMGv50, SMGvolume, PGmean, PGv30, PGv50, PGvolume, and weight loss rate were included in the LASSO method. Features with nonzero coefficients in the LASSO regression model were selected (16).

Multivariable logistic regression analysis was used to build a predicting model (model 1) by incorporating the features selected in the LASSO regression model. Another model (model 2) was conducted with the addition of SMGmean, SMGv50, and SMGvolume to the model 1. The incremental value of SMGmean, SMGv50, and SMGvolume as additional candidate predictors was calculated. C-index and calibration curve were derived. The net reclassification improvement (NRI) and the integrated discrimination improvement (IDI) were calculated (17, 18).

Backward step-wise selection was applied by using the likelihood ratio test with Akaike’s information criterion as the stopping rule (19). Calibration curves were plotted to assess the calibration of the nonadherence nomograms. A significant test statistic implies that the model does not calibrate perfectly (20). To quantify the discrimination performance of the nonadherence nomogram, Harrell’s C-index was measured. The nonadherence nomogram was subjected to bootstrapping validation (1,000 bootstrap resamples) to calculate a relatively corrected C-index (21).

Decision curve analysis was conducted to determine the clinical usefulness of the model 1 by quantifying the net benefits at different threshold probabilities (22). The decision curve was also plotted for the model 2 after the addition of SMGmean, SMGv50, and SMGvolume.

Statistical analyses were performed using SPSS Statistics Version 26.0 software (IBM Co., Armonk, NY, USA) and R software (version 3.6.2). Two-tailed *P*  values < 0.05 were considered statistically significant.

## Results

### Patient Characteristics

A total of 195 patients were included. The patient characteristics are showed in **Table 1**. Differences of SMGmean, SMGv50, SMGvolume, PGmean, PGv30, PGv50, PGvolume, and weight loss rate between grades 0–1 and grades 2–3 xerostomia are listed in **Table 2**.

**Table 1:** Patient characteristics.

variable	n
Age (years)	
Median	47
Range	15-74
Gender	
Male	144 (73.8%)
Female	51 (26.2%)
Pathology	
WHO II	24 (12.3%)
WHO III	171 (87.7%)
T stage	
T1	12 (6.2%)
T2	62 (31.8%)
T3	44 (22.6%)
T4	77 (39.4%)
N stage	
N0	11 (5.6%)
N1	80 (41.0%)
N2	79 (40.5%)
N3	25 (12.9%)
AJCC stage	
I	4 (2.0%)
II	38 (19.5%)
III	60 (30.8%)
IVa-b	93 (47.7%)
Chemotherapy	
No	20(10.3%)
Yes	175(89.7%)

WHO, World Health Organization; AJCC, the American Joint Committee on Cancer.

**Table 2:** Dosimetry parameters of submandibular glands and parotid glands on xerostomia at 12 months after treatment.

	Grade 0–1	Grade 2–3	P
SMGmean (Gy)	58.23 ± 6.26	59.13 ± 4.74	0.263
SMGv50 (%)	81.31 ± 18.80	83.85 ± 13.23	0.282
SMGvolume (cm^3^)	15.85 ± 4.76	14.92 ± 4.47	0.162
PGmean (Gy)	36.32 ± 2.63	38.81 ± 4.63	<0.001
PGV30 (%)	53.34 ± 6.21	58.77 ± 8.36	<0.001
PGv50 (%)	25.64 ± 5.64	29.72 ± 10.06	0.001
PGvolume (cm^3^)	59.62 ± 16.44	53.98 ± 15.68	0.015
Weight loss rate (%)	7.23 ± 4.56	8.24 ± 5.11	0.144

SMGmean, mean dose to the submandibular glands; SMGv50, V50 of the submandibular glands; SMGvolume, volume of the submandibular glands; PGmean, mean dose to the parotid glands; PGv30, V30 of the parotid glands; PGv50, V50 of the parotid glands; PGvolume, volume of the parotid glands.

### Predictors for Grades 2–3 Xerostomia

**Figure 1:**
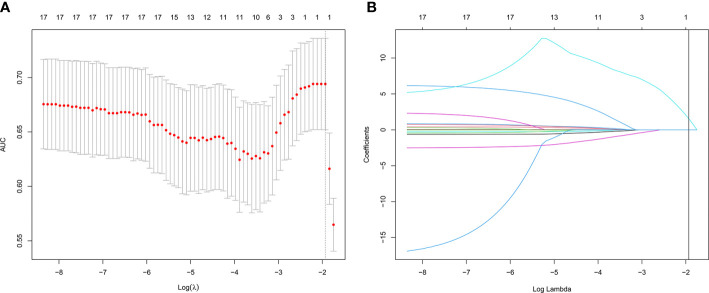
Texture feature selection using the least absolute shrinkage and selection operator (LASSO) binary logistic regression model. **(A)** Tuning parameter (λ) selection in the LASSO model used 10-fold cross-validation *via* minimum criteria. The area under the receiver operating characteristic (AUC) curve was plotted versus log (λ). Dotted vertical lines were drawn at the optimal values by using the minimum criteria and the 1 standard error of the minimum criteria (the 1-SE criteria). **(B)** LASSO coefficient profiles of the 13 texture features. A coefficient profile plot was produced against the log(lambda) sequence. Vertical line was drawn at the value selected using 10-fold cross-validation, where optimal lambda resulted in 1 nonzero coefficient.

Of the 13 features, one potential predictor (PGv30) was selected (**Figures 1A, B**), and were features with nonzero coefficients in the LASSO logistic regression model.

### Development of an Individualized Prediction Model

**Figure 2:**
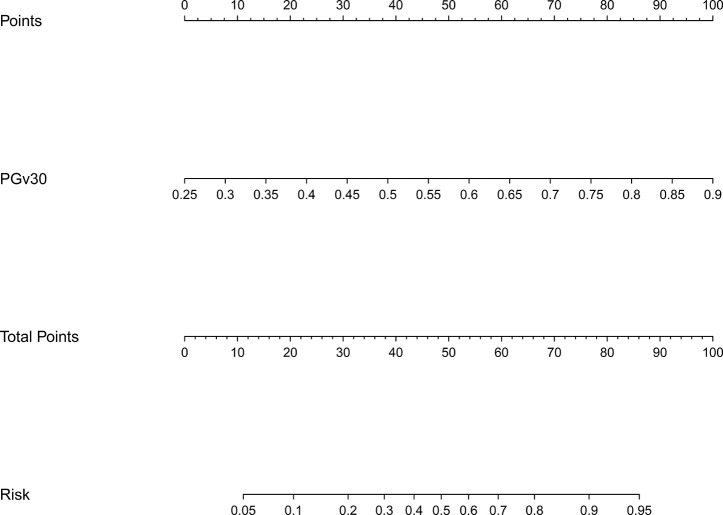
Nomogram of grades 2–3 xerostomia at 12 months after treatment (model 1). The nomogram was developed based on the result of the least absolute shrinkage and selection operator (LASSO) binary logistic regression model.

The model 1 that incorporated the feature selected in the LASSO regression model was developed and presented as the nomogram. The model 1 for grades 2–3 xerostomia at 12 months is showed in **Figure 2**.

### Apparent Performance of the Prediction Model

**Figure 3:**
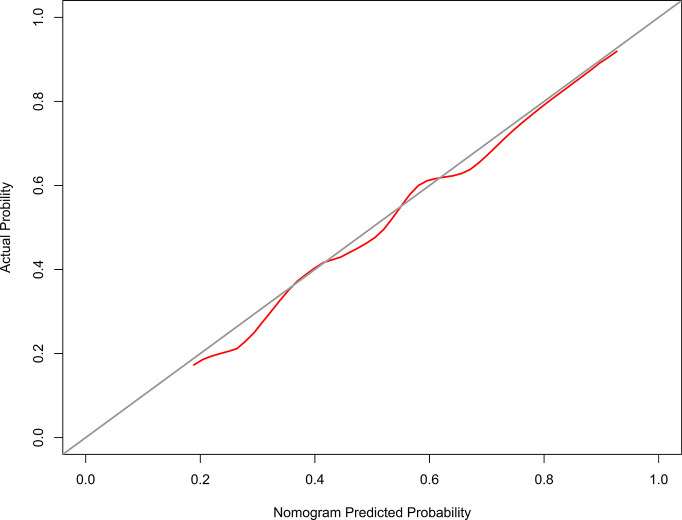
The Calibration curve of the nomogram for predicting grade 2–3 xerostomia at 12 months after treatment (model 1). The y-axis represents the actual grades 2–3 xerostomia rate. The x-axis represents the predicted grades 2–3 xerostomia risk. The diagonal line represents a perfect prediction by an ideal model. The red solid line represents the performance of the nomogram, of which a closer fit to the diagonal line represents a better prediction.

The calibration curve of the model 1 for the probability of grades 2–3 xerostomia demonstrated good agreement between prediction and observation (**Figure 3**). The C-index for the model 1 was 0.698 (95% confidence interval [CI]: 0.626–0.771).

### Incremental Predictive Value of Addition of the SMGmean, SMGv50, and SMGvolume

**Figure 4:**
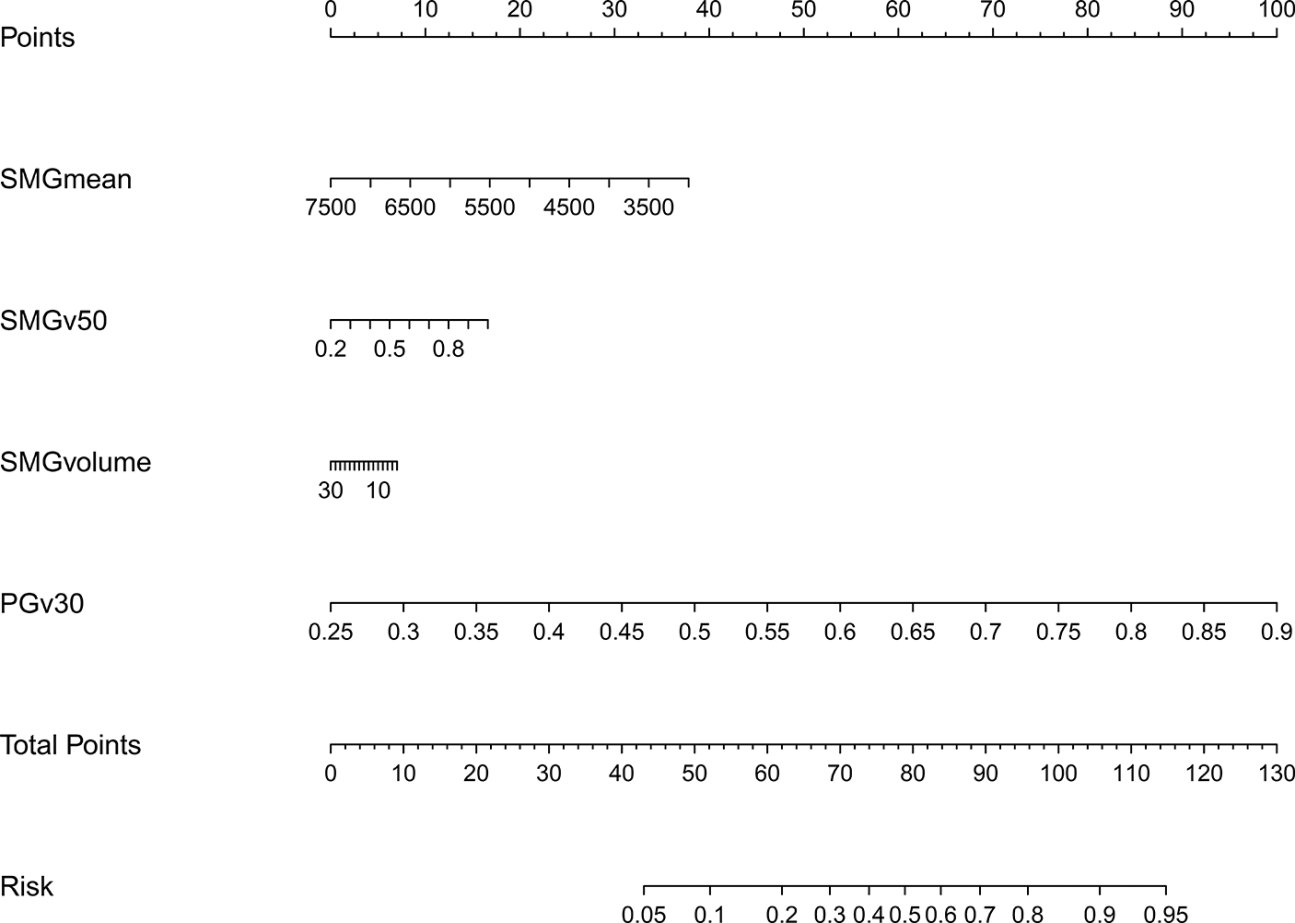
Nomogram of grades 2–3 xerostomia at 12 months after treatment (model 2). The nomogram was conducted with the addition of SMGmean, SMGv50, and SMGvolume to the model 1.

**Figure 5:**
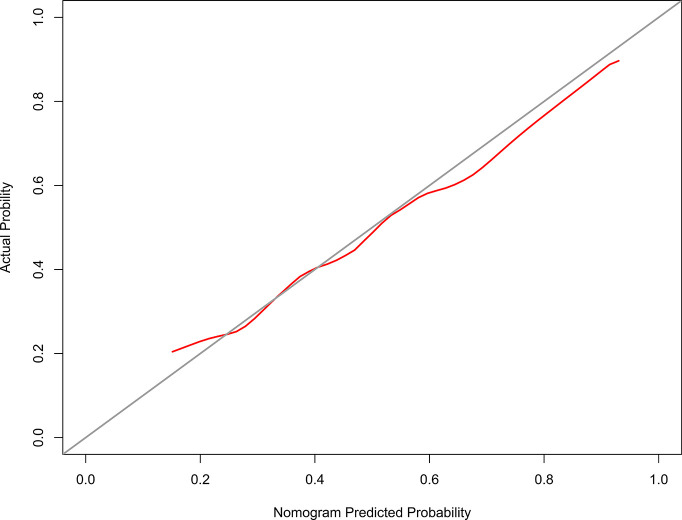
The Calibration curve of the nomogram for predicting grades 2–3 xerostomia at 12 months after treatment (model 2). The y-axis represents the actual grades 2–3 xerostomia rate. The x-axis represents the predicted grades 2–3 xerostomia risk. The diagonal line represents a perfect prediction by an ideal model. The red solid line represents the performance of the nomogram, of which a closer fit to the diagonal line represents a better prediction.

The model 2 that added the SMGmean, SMGv50, and SMGvolume to the model 1 was performed and presented as the nomogram. The model 2 for grades 2–3 xerostomia at 12 months is showed in **Figure 4**. The calibration curve of the model 2 for the probability of grades 2–3 xerostomia is showed in **Figure 5**. The C-index for the model 2 was 0.704 (95% CI: 0.632–0.776).

Although a slightly higher C-index was observed for the model 2, integration of the SMGmean, SMGv50, and SMGvolume into the model 1 did not show significantly improved prediction performance. NRI was 0.136 (95% CI: -0.144-0.416; *P* = 0.342). IDI was 0.008 (95% CI: -0.004–0.021; *P* = 0.168).

### Clinical Use

**Figure 6:**
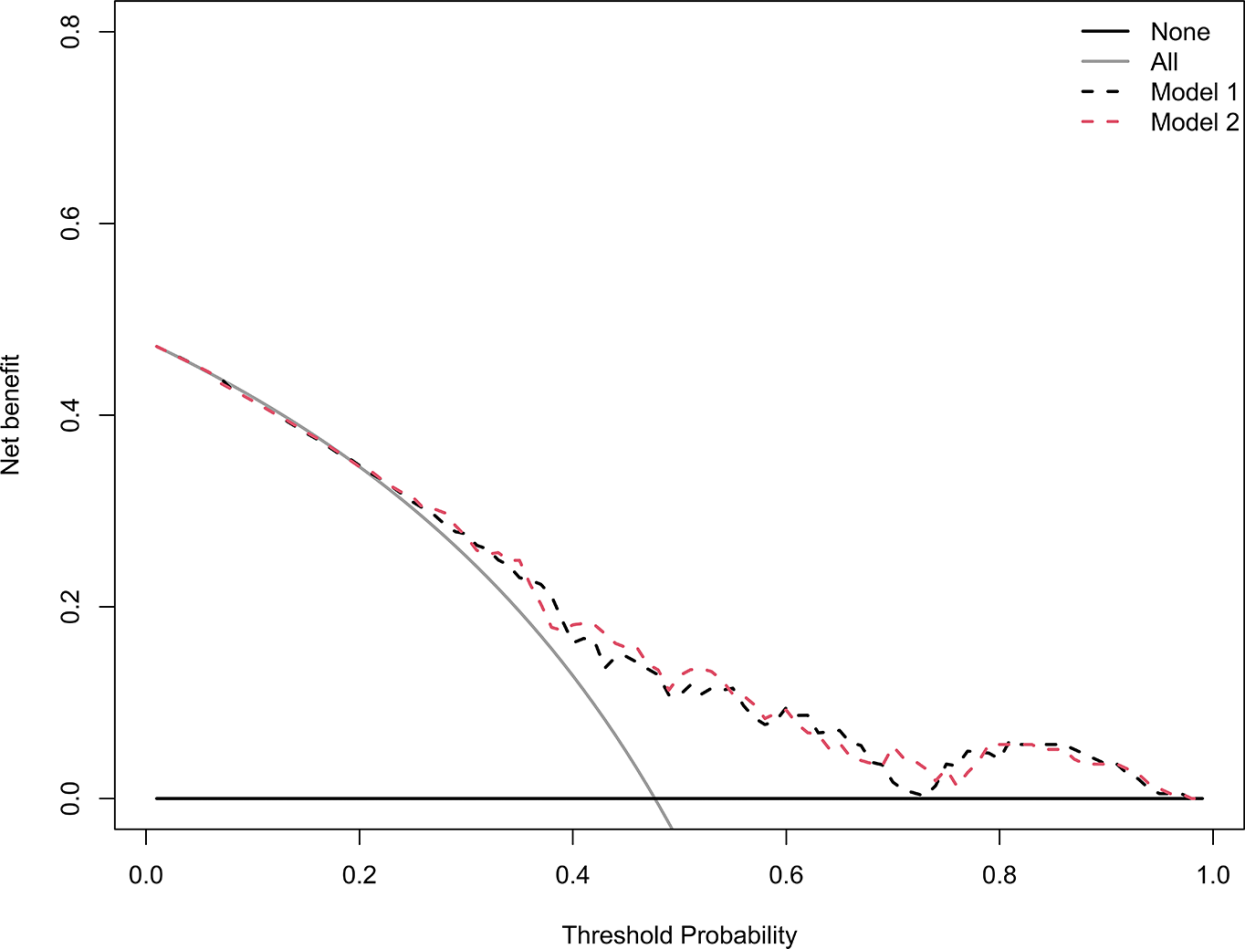
Decision curve analysis for the model 1 and the model 2. The y-axis measures the net benefit. The black dotted line represents the model 1. The red dotted line represents the model 2. The grey line represents the assumption that all patients have grades 2–3 xerostomia. Thin black solid line represents the assumption that no patients have grades 2–3 xerostomia. The net benefit was calculated by subtracting the proportion of all patients who are false positive from the proportion who are true positive, weighting by the relative harm of forgoing treatment compared with the negative consequences of an unnecessary treatment. The decision curve showed that if the threshold probability of a patient or doctor is > 20%, using the model 1 in the current study to predict grades 2–3 xerostomia adds more benefit than the treat-all-patients scheme or the treat-none scheme. Within this range, net benefit was comparable between the model 1 and model 2.

The decision curves analysis for model 1 and model 2 are presented in **Figure 6**. The decision curves showed that if the threshold probability of a patient or doctor is > 20%, using the model 1 to predict grades 2–3 xerostomia at 12 months after treatment adds more benefit than either the treat-all-patients scheme or the treat-none scheme. Within this range, net benefit was comparable between the model 1 and model 2.

## Discussion

This retrospective study indicated that PGv30 was an independent predictive factor of grades 2–3 xerostomia for NPC patients receiving IMRT. In contrast, the dosimetric parameters of submandibular glands including SMGmean, SMGv50, and SMGvolume were not independent predictive factors. Adding SMGmean, SMGv50, and SMGvolume to PGv30 did not provide a significantly improved predicted probability for grades 2–3 xerostomia.

Submandibular glands salivary flow rates depend on SMGmean. However, results on this issue were contradictory. It was reported that the submandibular glands salivary output recovered over time if SMGmean < 39 Gy in head and neck cancers (23). Grade of xerostomia would improve if SMGmean was reduced to below 39 Gy (24, 25). In contrast, several studies suggested that SMGmean was not correlated with patient’s self-reported xerostomia (26, 27). The differences between these studies arise possibly from the different study designs, including how salivary output was measured and small sample sizes. For NPC patients, Sommat et al. (28) reported that SMGmean was not associated with grade 2 and over salivary gland toxicity *via* physician-rated and patient-rated xerostomia. The present study observed a similar result that grades 2–3 xerostomia assessed according to RTOG/EORTC was not correlated with SMGmean of SMGv50.

Submandibular glands sparing was not performed in this study. Dose constraint of submandibular glands was not prescribed in IMRT plan. As a result, the median value of SMGmean was 58.96 Gy. Similarly, Wang et al. (25) reported a dose of 57.4 Gy in the non-submandibular glands sparing group. According to the dose-response relationships of the submandibular glands came from Tsujii et al. (29) salivary gland function improved as the dose increased from 10 to 30 Gy, followed by a steep decline after 50 Gy. Thus, the salivary output of submandibular glands was limited in this study. This might be a major reason for the negative result of the present study.

The main reason for non-submandibular glands sparing was that reduction of the radiation dose to the submandibular glands might be dangerous owing to its proximity to level II lymph nodes. However, Gensheimer et al. (24) reported that selected locally advanced oropharyngeal cancer with no definite contralateral neck disease were treated with submandibular glands-sparing IMRT. Submandibular glands-sparing IMRT did not increase marginal failures. Similarly, Wang et al. (25) revealed that no differences of overall survival (*P* < 0.05), local-regional-free survival (*P* < 0.05), and distant metastases-free survival (*P* < 0.05) were observed between submandibular glands sparing and non-submandibular glands sparing groups. Until now, evidence regarding the efficacy and safety of submandibular glands sparing in NPC is limited. Thus, when trying to preserve the function of the submandibular glands in NPC patients, physicians must consider the potential risk of reducing local regional tumour control.

All salivary glands should be assessed for xerostomia. Hawkins et al. (26) reported that combining doses to parotid glands, submandibular glands, and oral cavity yielded the highest marginal *R* ^2^ for xerostomia by comparison to models that included any one or combinations of any two structures. In our study, the oral cavity was not delineated as an organ at risk. It was not given dose constraint in designing the IMRT plan. However, our result indicated that adding SMGmean, SMGv50, and SMGvolume to PGv30 did not improve predicted probability. As a result, combination of PGv30, SMGmean and mean dose to oral cavity might not provide additional benefits. Possible reason for this hypothesis could be that minor salivary glands dispersed throughout the oral cavity only produce about 5% of salivary output (6, 7).

This study had a major limitation. Xerostomia was assessed according to the RTOG/EORTC system in this study (14). Patient’s self-reported xerostomia was not investigated. Because xerostomia is mainly an issue of quality of life. Patient’s subjective scores might be more reasonable endpoints in evaluating xerostomia (30). Comparing to the patient self-reported scores, the subjective assessment of the RTOG/EORTC system may underestimate the severity of xerostomia (28, 31). Thus, further studies are needed to verify the results of our study based on patient’s self-reported xerostomia.

In conclusion, this study suggested that SMGmean, SMGv50, and SMGvolume were not predictive factors of xerostomia in NPC patients receiving IMRT. Further studies of sparing submandibular glands are needed to verify the results of our study. Moreover, whether sparing submandibular glands is associated with increased risk of regional failure should be further investigated.

## Data Availability Statement

The raw data supporting the conclusions of this article will be made available by the authors, without undue reservation.

## Author Contributions

X-BP, YL, and X-DZ contributed to the conception of the study and performed the data analyses. S-TH, SP, and K-HC contributed to manuscript preparation. SQ and LL helped to perform the analysis with constructive discussions. All authors contributed to the article and approved the submitted version.

## Funding

This study was supported by the grant of Department of Education of Guangxi Zhuang Autonomous Region (no. KY2016LX029), the grant of Guangxi Medical University (no. GXMUYSF201521), the grant of Guangxi Health Committee (no. ZZ20200510), and the Research and Development Project of Guangxi (no. 1598012-22 and no. AB18221007).

## Conflict of Interest

The authors declare that the research was conducted in the absence of any commercial or financial relationships that could be construed as a potential conflict of interest.
